# Coevolution of competing *Callosobruchus* species does not stabilize coexistence

**DOI:** 10.1002/ece3.3003

**Published:** 2017-07-14

**Authors:** Stephen J. Hausch, Jeremy W. Fox, Steven M. Vamosi

**Affiliations:** ^1^ Department of Biological Sciences University of Calgary Calgary AB Canada

**Keywords:** *Callosobruchus chinensis*, *Callosobruchus maculatus*, coevolution, coexistence, competition, mutual invasibility

## Abstract

Interspecific resource competition is expected to select for divergence in resource use, weakening interspecific relative to intraspecific competition, thus promoting stable coexistence. More broadly, because interspecific competition reduces fitness, any mechanism of interspecific competition should generate selection favoring traits that weaken interspecific competition. However, species also can adapt to competition by increasing their competitive ability, potentially destabilizing coexistence. We reared two species of bean beetles, the specialist *Callosobruchus maculatus* and the generalist *C. chinensis*, in allopatry and sympatry on a mixture of adzuki beans and lentils, and assayed mutual invasibility after four, eight, and twelve generations of evolution. Contrary to the expectation that coevolution of competitors will weaken interspecific competition, the rate of mutual invasibility did not differ between sympatry and allopatry. Rather, the invasion rate of *C. chinensis*, but not *C. maculatus*, increased with duration of evolution, as *C. chinensis* adapted to lentils without experiencing reduced adaptation to adzuki beans, and regardless of the presence or absence of *C. maculatus*. Our results highlight that evolutionary responses to interspecific competition promote stable coexistence only under specific conditions that can be difficult to produce in practice.

## 1 INTRODUCTION

Stable species coexistence requires that every species be able to increase when rare (Chesson, 2000). An essential condition for each species to be able to increase when rare is that interspecific competition be weaker than intraspecific competition (Adler et al., 2007; Chesson, 2000). We expect this condition to hold because of evolution by natural selection: avoiding or reducing interspecific competition can increase fitness, and so selection should favor traits that weaken interspecific competition. Theory identifies conditions under which interspecific competition for shared limiting resources selects for the evolution of reduced resource use overlap (ecological character displacement; MacArthur & Levins, 1967; Slatkin, 1980; Abrams, 1986; Pfennig & Pfennig, 2010, 2012), thereby stabilizing coexistence (Lawlor & Maynard Smith, 1976). There are many putative examples of character displacement, although few definitive examples (Schluter, 2000; Stuart & Losos, 2013).

However, interspecific competition need not favor genotypes or phenotypes that reduce or avoid it (Aarssen, 1983). Instead, competition might favor increased tolerance to competition (Seaton & Antonovics, 1967) or increased competitive ability (e.g., Futuyma, 1970; Le Gac et al., 2012; Mitchell & Arthur, 1991; Nishikawa, 1985; Zhao et al., 2016). Even when interspecific competition favors character displacement or other mechanisms weakening interspecific relative to intraspecific competition, the net effect could still be to destabilize coexistence if selection also increases interspecific differences in competitive ability (Lankau, 2011). Conversely, if interspecific competition leads to evolutionary equalization of competitive ability, it might promote stable coexistence even if evolution does not weaken interspecific relative to intraspecific competition. Most theoretical models of character displacement ignore evolution of competitive ability, instead assuming that competing species of fixed (and sometimes equal) overall competitive ability use equally productive sets of resources (e.g., Abrams, 1986, 1987a,b; Lawlor & Maynard Smith, 1976; Slatkin, 1980; Taper & Case, 1992). Additionally, empirical studies rarely consider the question of how often interspecific competition promotes competitive coexistence. Natural examples of character displacement involve species that have co‐occurred for many generations and so likely are stably coexisting (Schluter & McPhail, 1992; Losos et al., 1998; Schluter, 2000; but see Grant & Grant, 2006). Cases in which interspecific competition and associated coevolution led to exclusion are difficult to study because they no longer exist. And experimental studies of the evolution of competitive ability are limited because they mostly consider (1) initially similar mutant and wild‐type strains of the same species, and (2) growth conditions that provide little to no opportunity for stable coexistence of different types (reviewed in Taper & Case, 1992). Accordingly, these experiments generally fail to find stable coexistence (reviewed in Taper & Case, 1992). More recent studies using different species and experimental designs often (but not always) fail to find that coevolution of competitors stabilizes their coexistence (Le Gac et al., 2012; Zuppinger‐Dingley et al. 2014, Maddamsetti et al., 2015; Zhao et al., 2016).

We tested whether interspecific competition promotes the evolution of stable coexistence by growing replicate populations of two competing species of bean beetles in allopatry and sympatry and assaying mutual invasibility after four, eight, or 12 generations of evolution. We used the competing bean beetles *Callosobruchus chinensis* and *C. maculatus*, reared on a mixture of two food resources (adzuki beans, *Vigna angularis*, and lentils, *Lens culinaris*). Bean beetles are a classic model system for studies of the eco‐evolutionary dynamics of competition (Smith, 1990; Taper, 1990; Utida, 1953). Their short generation time of ~23 days facilitates long‐term experiments. They reproduce sexually and adaptation depends primarily on standing genetic variation, making them a good match for both theoretical models of character displacement and natural systems in which character displacement has been found (Abrams, 1986; Stuart & Losos, 2013). Female bean beetles oviposit on the surface of beans into which the larvae burrow and develop, eventually emerging as sexually mature adults. Adults mate but do not feed, and die 4–8 days after emergence. Competition for resources occurs in two distinct but related stages: adults compete to claim resources by laying eggs on beans, with females preferring to avoid laying on beans on which eggs have already been laid. Larvae then compete with others in the same bean to acquire nutrients and develop faster (Vamosi, 2005). Providing two different resources (lentils and adzuki beans) provides an opportunity for evolutionary divergence in resource use, because the beetles exhibit an interspecific generalist‐specialist trade‐off in ability to use lentils and adzuki beans (they would not necessarily exhibit the same trade‐off if grown on other resources). Both species can consume adzuki beans, for which the specialist *C. maculatus* is the superior competitor (Hausch, 2015; Utida, 1953). The generalist *C. chinensis* also can consume lentils (*Lens culinaris*), on which *C. maculatus* larvae experience high mortality and slow development even in the absence of competition from other larvae (Hausch, 2015; Taper, 1990). Competition among larvae within beans is illustrated by per‐bean densities of eggs vs. emerging adults. Adzuki beans in established mixed cultures of *C. maculatus* and *C. chinensis* had an average of 48 ± 12 eggs/bean oviposited on them (mean ± *SE*), 89% of which hatched and burrowed into the bean but from which only six adults/bean emerged on average (S. Hausch, unpublished data). Lentils in established mixed cultures of *C. maculatus* and *C. chinensis* had an average of 14 ± 3 eggs/bean oviposited on them, 79% of which hatched and burrowed into the bean but from which only 1.5 adults/bean emerged on average (S. Hausch, unpublished data). Crucially, the number of adults emerging from a bean was independent of the number of eggs oviposited on the bean, implying that survival from egg to adult is inversely density dependent due to competitive interactions among larve within the same bean (S. Hausch, unpublished data).

If interspecific competition selects for divergence in resource use, or weakens interspecific competition via other mechanisms, then we expect more stable coexistence for sympatrically coevolved populations as competitors evolve to specialize on different resources. Specifically, we expect interspecific competition in sympatry to select for the generalist *C. chinensis* to specialize on lentils and for the specialist *C. maculatus* to increasingly specialize on adzuki beans. Conversely, lengthier allopatric evolution should weaken coexistence, as species evolve toward generalization, or else specialization on the same, productive resource. However, evolution of the competitive abilities of both species might either promote or inhibit coexistence. Taper (1990) selected the generalist *C. chinensis* to improve its ability to use lentils relative to its ability to use an initially preferred resource (mung beans), using both interspecific competition from *C. maculatus* and artificial selection against use of mung beans. Improvement in the ability of *C. chinensis* to use lentils did not come at a cost to its ability to use mung beans, and so presumably increased its overall competitive ability.

## 2 METHODS

### 2.1 Culture conditions

We obtained stock cultures from Dr. Yukihiko Toquenaga at the University of Tsukuba, Japan. Stock cultures were initiated from unique collection sites between 1940 and 2009 (Table 1) and have been maintained as unique lineages reared on adzuki beans. The stocks were chosen so as to provide substantial initial intraspecific variation within each species; evolution by natural selection would be ineffective without intraspecific variation to act on. For 1 year prior to this experiment, lineages were reared separately in clear 22 × 12 × 8 cm containers with fine mesh lids allowing gas exchange. Stock cultures were fed on a discrete schedule, receiving approximately 40 g of adzuki beans every 23 days to accommodate both early and late hatching lineages. Old beans and dead adults were removed approximately 14 days after feeding. Feeding schedules of stock cultures were initially synchronized through variation in rearing temperature. Stock cultures, treatments, and assays were all maintained in Percival I33LL environmental chambers at 30°C and 75% relative humidity in constant darkness. Temperature and humidity levels were chosen to equalize the developmental rates of the two species. Adzuki beans and lentils were purchased commercially, frozen at −18°C for 48 hr, rinsed, and finally dried at 35°C. Beans that were noticeably wrinkled or split were not used. Adzuki beans were used if they passed through a 6‐mm sieve but not a 3‐mm sieve.

**Table 1:** Name and origin of individual strains of *Callosobruchus* spp. “C” strains are *Callosobruchus chinensis*, “M” strains are *C. maculatus*

Strain	Strain name	Location of origin	Collection date
C1	Tsuchiura	Ibaraki, Japan	2009
C4	RDA MRKT	Rashahi, Bangladesh	1999
C5	jC	Kyoto, Japan	1940s
C6	Agrapathana	Colombo, Sri Lanka	2002
C7	sC bls	Nagano, Japan	1950s
M1	Oro Oro Dowo	Malang, Indonesia	2009
M2	Narahenpita National MRKT	Colombo, Sri Lanka	2002
M4	Nlongkak	Nlongkak, Cameroon	1998
M6	Indra Chowk	Kathmandu, Nepal	2005
M7	a°Q2	Unknown location, USA	1960s

### 2.2 Pre‐experiment preparations

Twenty populations of each of *C. maculatus* and *C. chinensis* were created by combining five conspecific lineages (Table 1). These lineages vary phenotypically when grown in the same environment and thus differ genetically (Mano & Toquenaga, 2008; Takano et al., 2001). In particular, whereas all lineages can exploit adzuki beans, only some lineages can exploit lentils. Lineages were raised to equilibrium densities on 5‐g adzuki beans. Mixing involved aggregating 20% of the infested beans from each lineage during the larval stage. Therefore, initial lineage frequencies were proportional to their adzuki bean carrying capacities. To allow some genetic mixing, the high‐diversity populations were fed 5‐g adzuki beans and 5‐g lentils for one generation prior to initiating the treatments. At the end of this generation, emerging adults were enumerated daily, recording their species identity and the resource from which they emerged.

### 2.3 Experimental design overview

**Figure 1:**
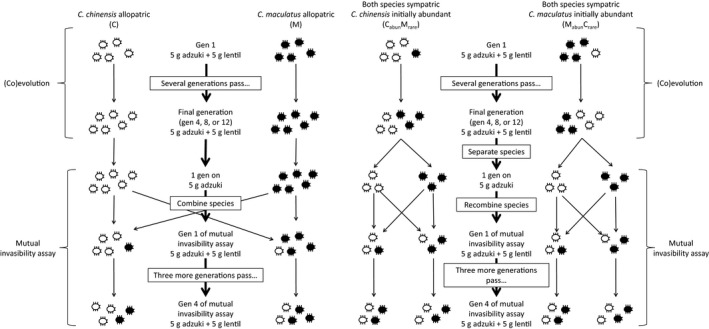
Schematic of the experiment. White beetles represent *C. chinensis*, filled beetles represent *C. maculatus*. Numbers of beetles are not indicative of actual densities in the experiment

We first provide an overview of our design (Figure 1), deferring technical details to the following subsection.

We randomly assigned the 40 populations to four treatments with 10 replicates each: (1) M—*C. maculatus* alone, (2) C—*C. chinensis* alone, (3) M_abun_C_rare_—*C. maculatus* initially abundant, *C. chinensis* initially rare, and (4) C_abun_M_rare_—*C. chinensis* initially abundant, *C. maculatus* initially rare. Each species thus experienced three treatments: allopatric and two sympatric treatments (initially abundant and initially rare). We included two sympatric treatments because the long‐term outcome of competitive coevolution might be sensitive to species’ initial abundances when they first come into sympatry, for instance because of founder effects and drift in initially small populations. In practice, the two sympatric treatments differed from one another only in the short term, see section 3.

We also wanted to test for differences between short‐ and longer‐term effects of (co)evolution. We therefore randomly chose three replicates of each treatment to assay for strength of coexistence after four generations of (co)evolution, tree replicates to assay after eight generations, and assayed the remaining four replicates after 12 generations. Each generation lasted 23 days. Assaying strength of coexistence was a destructive process, so once a replicate was assayed, it could not be assayed again.

### 2.4 Experimental design details

To start the experiment, we randomly assigned 10 populations of each species to grow allopatrically. Each of the other 10 populations of each species was divided into two subpopulations, comprising four (~15%) of the infested adzuki beans, and the remaining ~85% of the infested adzuki beans. Replicates of the M_abun_C_rare_ treatment were produced by combining a small (four bean) subpopulation of *C. chinensis* with a randomly chosen large subpopulation of *C. maculatus*. Replicates of the C_abun_M_rare_ treatment were produced by combining a small (four bean) subpopulation of *C. maculatus* with a randomly chosen large subpopulation of *C. chinensis*. Initially rare sympatric populations thus began at ~15% of carrying capacity, whereas initially common sympatric populations began at ~85% of carrying capacity.

For the duration of the experiment, communities were reared in 10‐cm diameter, quarter‐partitioned petri dishes under the same temperature, humidity, and light conditions as the stock cultures. Beetles were fed 5‐g adzuki beans and 5‐g lentils. Beetles were not confined to a single partition. The purpose of the quarters was to separate each generation of beans, so that we could identify if beetles were emerging from this generation's or a previous generation's beans, while keeping all beans available for use. On the first day of each generation, fresh beans were added into an unfilled quarter of the petri dish. On day 18 of each generation, dead adults were removed, identified to species, and enumerated. On the 18th day of generations four, eight, and 12, infested lentils and adzuki beans were separated by bean species. Emerging adults from each bean species were identified and enumerated daily, then either used to initiate mutual invasibility assays, or else re‐aggregated into a single community.

### 2.5 Mutual invasibility (strength of coexistence) assays

Mutual invasibility was assayed after four, eight, or 12 generations. Prior to assaying mutual invasibility, emerging adults from the fourth, eighth, or twelfth generation were identified and enumerated daily and the species were separated for one generation. While separated, species were provided only 5 g of adzuki beans (i.e., no lentils) to reduce the possibility that resource partitioning in the mutual invasibility assays was a plastic response to the recent availability of two bean species. Seventeen days after feeding with 5‐g adzuki beans, dead adults were removed and the assays were initiated by exchanging four (~15%) of the infested adzuki beans between heterospecific population pairs, as in the main experiment. The mutual invasibility assays were maintained for three more generations, under the same conditions as the main experiment, with the number of adults enumerated after each generation had died (Figs. S2, S3). Three generations was sufficient for most populations to approach or surpass the initial density of the resident population (see section****** 3). Before emergence of the fourth and final generation, the adzuki beans and lentils were separated. The adults emerging from each resource were identified and enumerated daily.

### 2.6 Statistical analyses

Invasion rate was estimated using nonlinear regression, fitting the invasion dynamics to

$$N=\left[\frac{\left(1+h\right)\times x}{1+h\times x}\times k\right]+\text{initial}$$

where *N* is population density; *k* is the increase in density over the four generations; initial is the starting density of the invader (taken from census data); *x* is time since the beginning of a mutual invasibility assay, scaled between zero and one; and *h* controls the curvature of the change in density during the four generations. As *x* goes to 1, the final density equals initial + *k*. When *h *=* *0, growth is linear. If *k *>* *0, positive values of *h* represent decelerating, asymptotic growth, whereas negative values (but >−1) represent accelerating growth. We defined invasion rate *I* as the predicted increase in *N* from *initial* until *x *=* *0.33 (i.e., after one generation),

$$I=\left[\frac{\left(1+h\right)0.33}{1+0.33h}k\right]$$

Use of this metric assumes that the density of the resident population was constant across generations and treatments. This assumption was approximately satisfied: Resident population densities were relatively constant, except for rapid increases if the invader failed to increase (median CV = 0.23; see section 3). In one case, *C. chinensis* had been competitively excluded during experimental evolution, so this replicate (assayed in generation eight) was excluded from the analysis.

We used the invasion‐rate metric *I* to test for treatment and phenotype effects on the stability of coexistence. The effects of evolutionary treatment (allopatric, sympatric with *C. chinensis* initially abundant, and sympatric with *C. maculatus* initially abundant) and duration (four, eight, or 12 generations of evolution before mutual invasibility was assayed) on invasion rate were analyzed using ANOVA with planned contrasts. Eight planned contrasts were applied: the six contrasts between the sympatric and allopatric populations within a duration (nonorthogonal); and two contrasts of four vs. eight generations of evolution, and eight vs. 12 generations.

The relative densities emerging from each resource reveals insight into the underlying mechanisms by which coevolution affects competitive coexistence. In any system in which competition occurs within different spatial “niches” (here, within beans), with each niche contributing some proportion of offspring to a common global pool, coexistence is most stable when each competing type has a niche in which its fitness greatly exceeds that of competing types (Kassen et al., 2000; Levene, 1953). If interspecific competition promotes coexistence by selecting for resource partitioning, then when both species coevolve in sympatry each should become increasingly dominant on the resource on which it has a competitive advantage (adzuki beans for the specialist *C. maculatus*, lentils for the generalist *C. chinensis*). This could come about through evolutionary shifts in egg‐laying preferences, in larval performance, or both. We used our data on larval emergence from beans at the end of each mutual invasibility assay to estimate the dominance of *C. chinensis* on lentils, and of *C. maculatus* on adzuki beans. We estimated the dominance of *C. chinensis* on lentils as the proportion of *C. chinensis* among all individuals emerging from lentils at the end of the mutual invasibility assay and did the same for *C. maculatus* on adzuki beans. We calculated *C. chinensis* dominance of lentils only for those mutual invasibility assays in which *C. chinensis* was the invader, and similarly for *C. maculatus* dominance of adzuki beans. The initially high abundance of the resident species in the mutual invasibility assays effectively guarantees that, even after four generations, almost 100% of the individuals emerging from the resource on which the resident is the competitive dominant will be members of the resident species, regardless of evolutionary history. Logit transformed dominance of lentils by *C. chinensis*, and of adzuki beans by *C. maculatus*, was analyzed using the same approach as for invasion rate. However, because both species will use both resources to varying degrees, only replicates where both species reached a final density of greater than 50 individuals were considered (i.e., six replicates were excluded).

All statistical analyses were conducted in R (R Core Team 2015).

### 2.7 The potential for other mechanisms of competition

We observed only scramble larval competition during our experiments, although contest competition (aggression against other larvae within the same bean) is well documented in other studies of *Callosobruchus* spp. (Toquenaga, 1993). Standard assays for contest competition, and X‐rays of beans, failed to identify any evidence for contest competition among our beetles (Hausch, 2015). The two species may also compete intra‐ and interspecifically via other mechanisms, such as egg mortality caused by high densities of adults damaging eggs as they move over bean surfaces (Fujii, 2009), and mating interference of *C. chinensis* males with *C. maculatus* females (Kishi & Tsubaki, 2014). Mutual invasibility, or the lack thereof, represents the net outcome of all mechanisms by which the two beetle species interact intra‐ and interspecifically. We focus in the first instance on the evolution of mutual invasibility. The underlying biological mechanisms affect species coexistence only via their effects on mutual invasibility. Further, all underlying mechanisms of interspecific competition should select for traits that avoid competition and/or traits that improve competitive ability.

### 2.8 Data availability

Data available from the Dryad Digital Repository: http://dx.doi.org/10.5061/dryad.70057.

## 3 RESULTS

*C. chinensis* and *C. maculatus* competed with one another. After 12 generations, *C. chinensis* and *C. maculatus* attained abundances of 519 ± 20 and 465 ± 24 individuals, respectively, when growing in allopatry (mean ± *SD*). *C. chinensis* and *C. maculatus* attained reduced abundances of 259 ± 36 and 210 ± 30, respectively, when growing in sympatry (combined data from both sympatric treatments).

**Figure 2:**
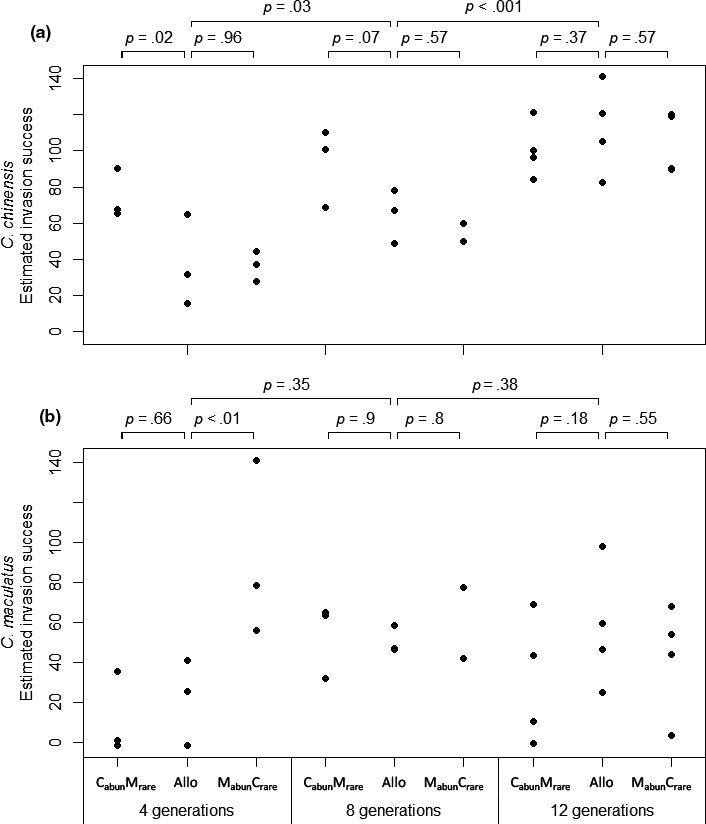
Estimated invasion success of *C. chinensis* (a) and *C. maculatus* (b) populations, across time and treatments. Each point represents data from one population.Invasion rate was estimated as the predicted density one generation after invading using a curve fit to the population trajectory (see section 2). Bars above the plot denote planned contrasts and their *p* values. The two wider bars at the top are contrasts between durations of evolution, averaging across the three treatments within a duration. Allo, allopatric; C_abun_M_rare_, sympatric with *C. chinensis* initially abundant; M_abun_C_rare_, sympatric with *C. maculatus* initially abundant.

Most population pairs were mutually invasible, but with variation in the rates of invasion. The invasion rate of the generalist *C. chinensis* varied significantly among treatment combinations (ANOVA, *F*_8,20_ = 8.3, *p *<* *.001; Figure 2a). *C. chinensis* invaded faster when initially common and grown in sympatry with *C. maculatus* (C_abun_M_rare_) than in the allopatric control after coevolving for four and eight generations, although the latter difference is marginally nonsignificant (planned contrasts, Figure 2a). *C. chinensis* invasion rate in the other sympatric treatment **(**M_abun_C_rare_ **)** did not differ significantly from the allopatric control (planned contrasts, Figure 2a). The average invasion rate of *C. chinensis* increased significantly from four to eight generations and from eight to 12 generations (planned contrasts, Figure 2a). Both of these increases occurred primarily in the allopatric and M_abun_C_rare_ treatments (Figure 2a). Thus, after 12 generations of evolution, the invasion rate of *C. chinensis* that had evolved in sympatry did not differ significantly from those that evolved in allopatry (planned contrasts, Figure 2a).

The invasion rate of the specialist *C. maculatus* did not vary significantly among treatments (ANOVA, *F*_8,20_ = 2.3, *p *=* *.066; Figure 2b). In planned contrasts, the invasion rate of *C. maculatus* was higher when it was sympatric with *C. chinensis* and initially common (M_abun_C_rare_) relative to the allopatric control after four generations of coevolution (planned contrast, Figure 2b).

**Figure 3:**
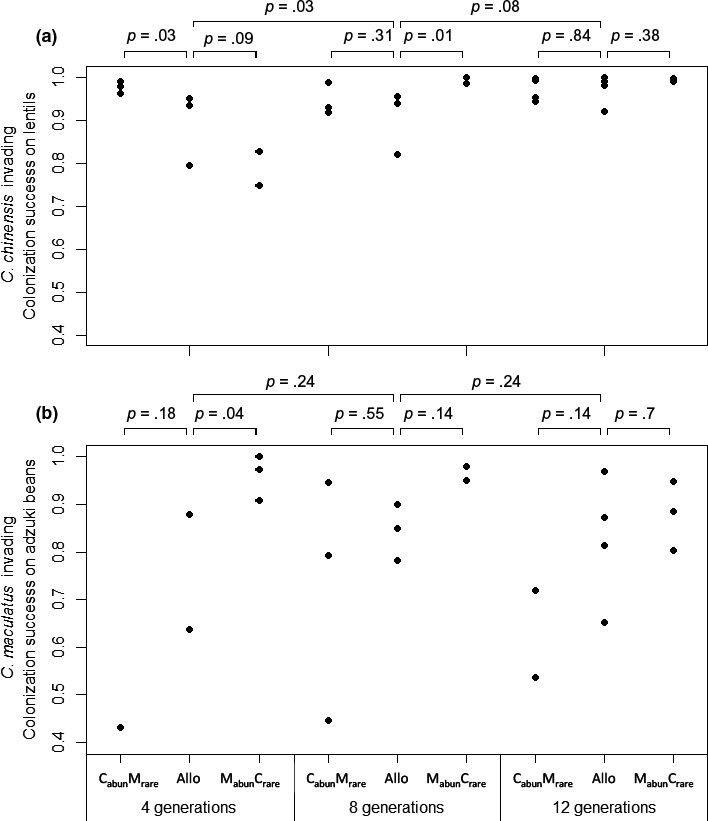
Realized resource partitioning in the mutual invasibility assays with *C. chinensis* (a) or *C. maculatus* (b) invading, across time and treatments.Bars above the plot denote planned contrasts and their *p* values. The two wider bars at the top are contrasts between durations of evolution, averaging across the three treatments within a duration. Allo, allopatric; C_abun_M_rare_, sympatric with *C. chinensis* initially abundant; M_abun_C_rare_, sympatric with *C. maculatus* initially abundant.

As expected, the generalist *C. chinensis* emerged primarily from lentils, whereas the specialist *C. maculatus* emerged primarily from adzuki beans: across all mutual invasibility assays, a median of 89% of invading *C. chinensis* emerged from lentils and 98% of invading *C. maculatus* emerged from adzuki beans. *C. chinensis* emerged primarily from lentils and *C. maculatus* emerged primarily from adzuki beans even though both species continued to lay eggs on both bean species throughout the experiment (S. Hausch, personal observation). Realized resource partitioning therefore reflected the outcome of interspecific competition among larvae within beans, rather than avoidance of interspecific competition at the egg laying stage. However, there was no significant variation among treatments in the ability of *C. chinensis* to dominate lentils, or the ability of *C. maculatus* to dominate adzuki beans, save for early in the experiment. After four generations, *C. chinensis* dominated lentils and *C. maculatus* dominated adzuki beans more after evolving as initial residents in sympatry than in allopatry (planned contrasts, Figure 3), but these differences were no longer significant by generation 12. Rather than varying among treatments, dominance of lentils by *C. chinensis* increased significantly from generations four to eight, and marginally nonsignificantly from generations eight to 12 (planned contrasts, Figure 3a).

Population dynamics from the main experiment, and from all of the mutual invasibility assays, are shown in Figs. S1–S3.

## 4 DISCUSSION

The strength of mutual invasibility did not increase when competing species coevolved in sympatry rather than evolving in allopatry, even though the experiment was explicitly designed to give it every opportunity to do so. Instead, *C. chinensis* evolving in both allopatry and sympatry became better able to invade *C. maculatus* populations (increase in average *I*), without a corresponding reduction in ability to repel invasion by *C. maculatus*. The strength of mutual invasibility thus increased over time, but not because of coevolution between the competing species. Rather, both sympatric and allopatric evolution improved *C. chinensis*’ dominance of lentils, but not *C. maculatus*’ dominance of adzuki beans.

Interspecific competition apparently did not alter the direction of selection (termed “neighbor‐dependent selection” by Vasseur et al., 2011). Rather, interspecific competition apparently altered the strength and efficiency of selection early in the experiment. The efficiency of selection likely was reduced when populations began coevolution as initially rare invaders, due to reduced population size early in the experiment. The strength of selection likely was reduced when populations evolved in allopatry due to reduced competition. Because of stronger, more efficient selection, initially common resident sympatric populations rapidly evolved higher invasion rates than other populations after four generations. They also evolved stronger dominance of their “own” resource. After this time, sympatric populations achieved similar densities independent of whether they were initially common or initially rare, reducing variation in the efficiency of selection across treatments. Invasion rates and resource dominance of other populations eventually caught up to those of the initially common sympatric populations, the latter perhaps having approached an adaptive peak.

*Callosobruchus chinensis* adapted to the novel resource, lentils, rather than to its competitor *C. maculatus*. If evolution had been dominated by adaptation to interspecific competition, sympatric initially rare invader populations would have evolved the fastest invasion rates, because initially rare populations necessarily experience primarily interspecific rather than intraspecific competition until they become sufficiently abundant (Pimentel et al., 1965; Vasseur et al., 2011). This finding is consistent with experimental evolution in *Drosophila* spp. (Futuyma, 1970; Joshi & Thompson, 1996). When *Drosophila* populations were presented simultaneously with a novel competitor and a novel resource, evolution was dominated by adaptation to the novel resource (Joshi & Thompson, 1996).

Our results are consistent with empirical studies of the evolution of competitive ability, which find that competitive coevolution generally does not promote coexistence (reviewed in Taper & Case, 1992). A few studies find stronger coexistence in coevolved communities (Maddamsetti et al., 2015; Martin & Harding, 1981; Seaton & Antonovics, 1967; Turkington & Harper, 1979). However, most studies find that competitive ability either does not evolve or else does not evolve in such a way as to promote stable coexistence (Dawson, 1972; Futuyma, 1970; Hedrick, 1972; Mitchell & Arthur, 1991; Park & Lloyd, 1955; Sokal et al., 1970; Sulzbach, 1980; Sulzbach & Emlen, 1979). Failure of coexistence to evolve in many of these studies might reflect lack of opportunity for coexistence to evolve (e.g., provision of only one food resource). Competitive coevolution also can fail to maintain or increase the stability of pre‐existing stable coexistence. Le Gac et al. (2012) tracked the eco‐evolutionary dynamics of two competing *E. coli* lineages that stably coexisted for over 30,000 generations. Both lineages competed for glucose, with the subordinate lineage persisting via cross‐feeding (consuming a metabolic byproduct secreted by the dominant lineage). Over time, the dominant lineage evolved so as to encroach on the resource use niche of the subordinate lineage and would have excluded it had the subordinate lineage not continued to evolve increased competitive ability (Le Gac et al., 2012). Similarly, Maddamsetti et al. (2015) reported stable coexistence of two competing *E. coli* lineages for >6,000 generations, before the accumulation of beneficial mutations in one lineage allowed it to outcompete the other. Zhao et al. (2016) reported that sympatric coevolution of pairs of *E. coli* lineages for 1,100 generations did not reduce niche overlap or further stabilize coexistence.

It would have been challenging but interesting to run our experiment for many more generations, so as to test whether in the long run, *C. chinensis* would evolve so as to further encroach on the niche of *C. maculatus*, possibly excluding it. This is the long‐term outcome that theory would predict. When trade‐offs in the use of nutritionally substitutable resources are weak, as they apparently are for *C. chinensis* (Taper, 1990), theory predicts the evolution and competitive dominance of generalists over specialists (Levins, 1962; Rueffler et al., 2006; Wilson & Turelli, 1986). Evolutionary increases in the absolute number of *C. chinensis* emerging from lentils, a resource that *C. chinensis* dominates almost completely, would eventually allow it to swamp *C. maculatus*, unless that species were able to evolve increased dominance of adzuki beans (Kassen et al., 2000; Levene, 1953).

Further work would be needed to fully describe the biological mechanisms underlying our results. Improved larval survival and development, higher adult fecundity, stronger mating interference, and other biological mechanisms could contribute to the evolutionary improvement in *C. chinensis*’ ability to invade *C. maculatus*. However, the underlying mechanisms of improvement must be those that are equally favored by selection in sympatry and allopatry, which suggests that evolved changes in interspecific mating interference are not the main driver of our results. Any direct selection on mating interference by *C. chinensis*, and on the ability of *C. maculatus* to avoid or overcome mating interference by *C. chinensis*, should only occur in sympatry.

The theoretical prediction that coevolution among competitors will stabilize coexistence by increasing niche differentiation depends on numerous strong assumptions: competing species of equal, unchanging overall competitive ability, using equally productive ranges of resources, and subject to strong trade‐offs such that evolving increased competitive ability for one resource accompanies the evolution of decreased competitive ability for another. Our results highlight that real organisms often violate some or all of these assumptions, and when they do coevolution of competitors will not stabilize coexistence (Abrams, 1986). However, the relative paucity of empirical studies of competitive coevolution, and the limited range of species and culture conditions used by those studies, makes it difficult to say whether competitive coevolution typically promotes stable coexistence. Future theoretical and empirical work on the ecological consequences of competitive coevolution should investigate the evolution of equalizing mechanisms as well as stabilizing mechanisms.

## CONFLICT OF INTEREST

None declared.
