# Supplementary material for: Coevolution of competing Callosobruchus species does not stabilize coexistence
Source: Ecol Evol. 2017 Jul 14;7(16):6540–8. doi: 10.1002/ece3.3003 (PMC5574802; doi:10.1002/ece3.3003)
Supplement: Supplementary file 1 [file ECE3-7-6540-s001.docx]

**Fig. S1:** Population dynamics of *C. chinensis* and *C. maculatus* in the mutual invasibility assays.

Panels A and B – allopatric; C and D – sympatric with *C. chinensis* initially abundant; E and F – sympatric with *C. maculatus* initially abundant. Panels A, C, and E – *C. maculatus* invading; B, D, and F – *C. chinensis* invading. Common line style within a treatment (solid, dashed, dotted, or dash-dotted) signifies population pairs: the invader population with a given line style invaded the resident population with that line style.
