# Supplementary material for: Coevolution of competing Callosobruchus species does not stabilize coexistence
Source: Ecol Evol. 2017 Jul 14;7(16):6540–8. doi: 10.1002/ece3.3003 (PMC5574802; doi:10.1002/ece3.3003)

Fig. S2: Frequency of species in sympatric evolution treatments and associated mutual invasibility assays.

Populations move down the y-axis through time with shifts along the x-axis signifying invasions (at generation 0 to start the experiment, and at generation 4, 8, or 12 to assay mutual invasibility). Dashed lines separate replicates. The fill of the cell at each point in time represents the portion of the community that is *C. maculatus* (dark) and *C. chinensis* (light). For each replicate, the left stack of cells is the M_abun_C_rare_ treatment and the right stack of cells is the C_abun_M_rare_ treatment. Unfilled cells signify generations during which abundances were not tracked.


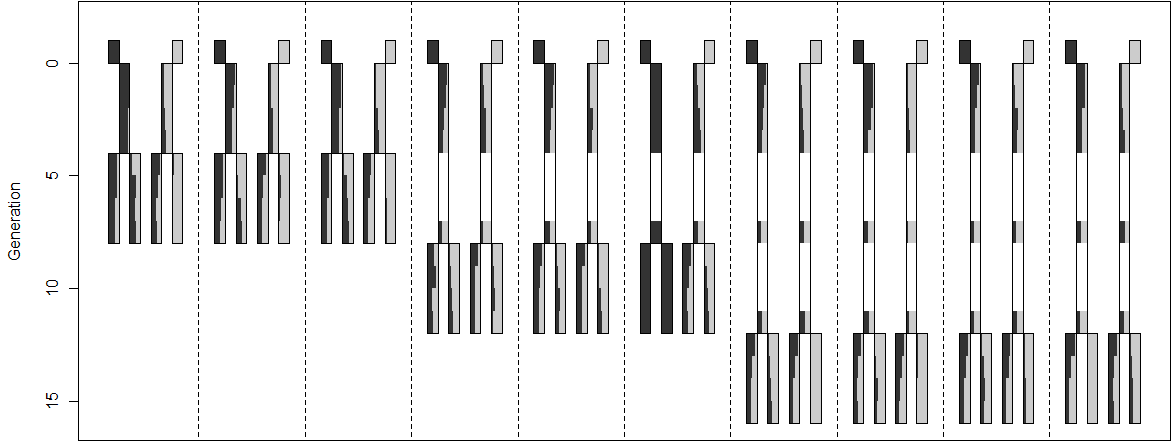

Supplement: Supplementary file 2 [file ECE3-7-6540-s002.docx]
